# Supplementary material for: Dynamics of Microorganisms and Metabolites in the Mixed Silage of Oats and Vetch in Alpine Pastures, and Their Regulatory Mechanisms Under Low Temperatures
Source: Microorganisms. 2025 Jun 30;13(7):1535. doi: 10.3390/microorganisms13071535 (PMC12300317; doi:10.3390/microorganisms13071535)
Supplement: Supplementary file 1 [file microorganisms-13-01535-s001.zip › Supplementary Table.pdf]

Supplementary Table S1 Bacterial high-throughput sequencing data for oat and vetch silage mixes

Sample ID	Raw CCS	Clean CCS	Effective CCS
CK0d 1	10211	10208	10064
CK0d 2	18260	18252	17771
CK0d 3	16606	16600	16306
CK0d 4	18220	18213	17809
CK0d 5	13352	13347	13221
CK0d 6	8226	8222	8194
CK90d 1	16505	16497	16000
CK90d 2	18696	18683	16628
CK90d 3	9365	9358	9064
CK90d 4	8225	8211	7850
CK90d 5	15310	15309	15193
CK90d 6	14698	14696	14591
aggregate total	167674	167596	162691

Supplementary Table S2 Statistics on the diversity of bacterial communities in oat and vetch silage mixes

sample	Kindom	Phylum	Class	Order	Family	Genus	Species
CK0d 1	1	10	14	38	59	97	133
CK0d 2	1	11	15	34	62	119	174
CK0d 3	1	14	20	47	89	155	214
CK0d 4	1	14	20	41	75	160	227
CK0d 5	1	9	13	33	57	97	123
CK0d 6	1	8	12	25	41	76	96
CK90d 1	1	11	16	36	72	141	218
CK90d 2	1	17	24	51	94	182	254
CK90d 3	2	11	15	35	65	123	178
CK90d 4	1	14	21	42	80	159	230
CK90d 5	2	14	19	42	78	149	206
CK90d 6	2	12	17	36	68	125	177

Supplementary Table S3 High-throughput sequencing data on fungi of oat and vetch silage mixes

Sample ID	Raw CCS	Clean CCS	Effective CCS
CK0d 1	12347	12345	10818
CK0d 2	14100	14096	13417
CK0d 3	9276	9276	8905
CK0d 4	12191	12190	11511
CK0d 5	11722	11721	11310
CK0d 6	13797	13794	13219
CK90d 1	12557	12556	12412
CK90d 2	17991	17990	17773
CK90d 3	8146	8146	8088
CK90d 4	10375	10373	10227
CK90d 5	12836	12832	12633
CK90d 6	13020	13020	12856
aggregate total	148358	148339	143169

Supplementary Table S4 Statistics on the diversity of fungal communities in oat and vetch silage mixes

Sample	Kindom	Phylum	Class	Order	Family	Genus	Species
A1	1	3	17	35	52	76	103
A2	1	7	27	52	92	134	167
A3	1	4	14	25	35	53	70
A4	1	3	15	33	48	68	90
A5	1	4	14	31	46	65	87
A6	1	4	15	29	37	61	76
B1	1	7	17	28	42	53	61
B2	1	8	25	45	69	105	123
B3	1	3	10	23	33	45	49
B4	1	7	17	27	39	56	69
B5	1	7	17	27	37	53	60
B6	1	3	11	23	35	46	57
