# Supplementary figures and images for: Dynamics of Microorganisms and Metabolites in the Mixed Silage of Oats and Vetch in Alpine Pastures, and Their Regulatory Mechanisms Under Low Temperatures
Source: Microorganisms. 2025 Jun 30;13(7):1535. doi: 10.3390/microorganisms13071535 (PMC12300317; doi:10.3390/microorganisms13071535)

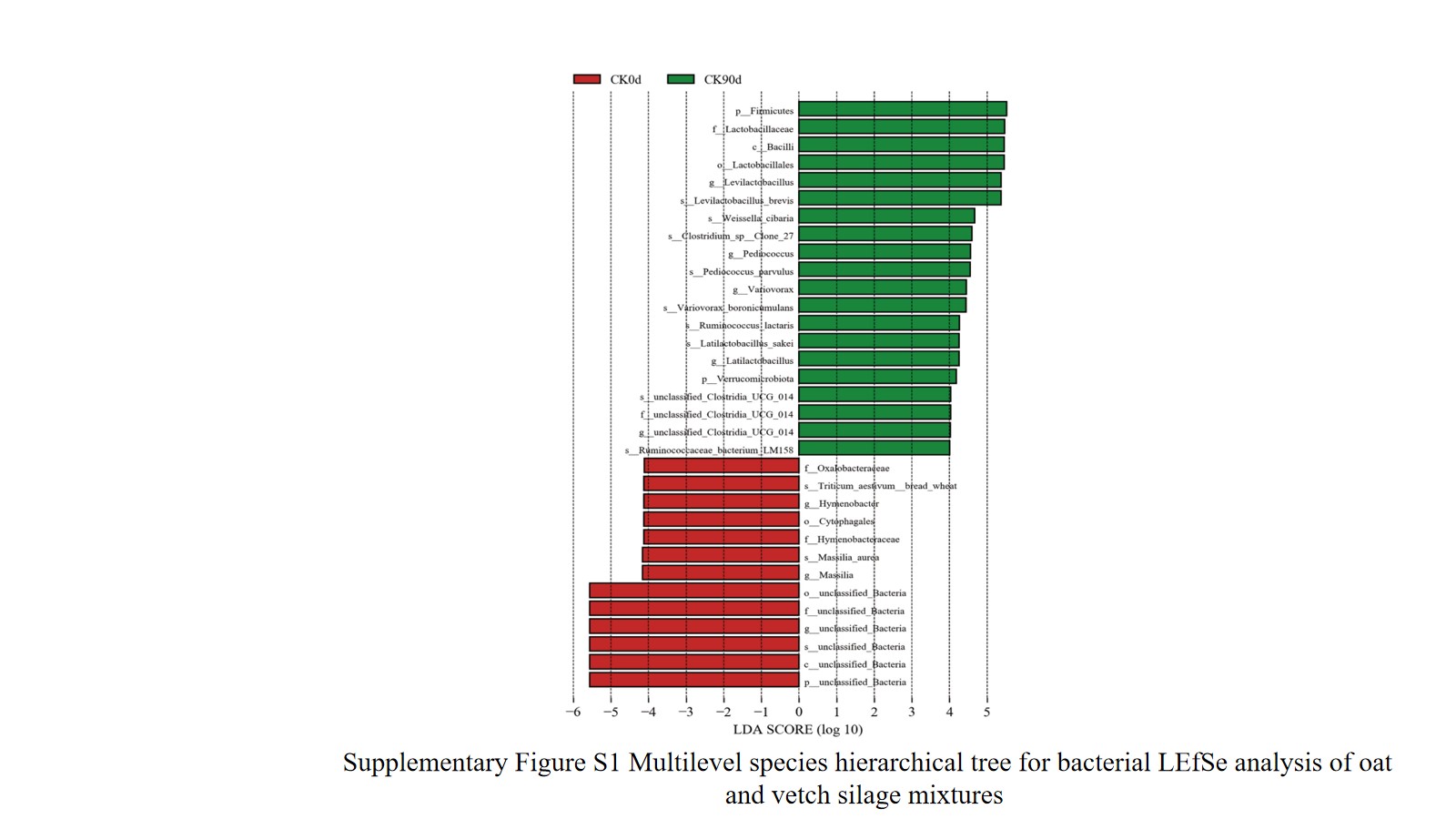

Supplement: Supplementary file 1 [file microorganisms-13-01535-s001.zip › Supplementary Figure S1.jpg]

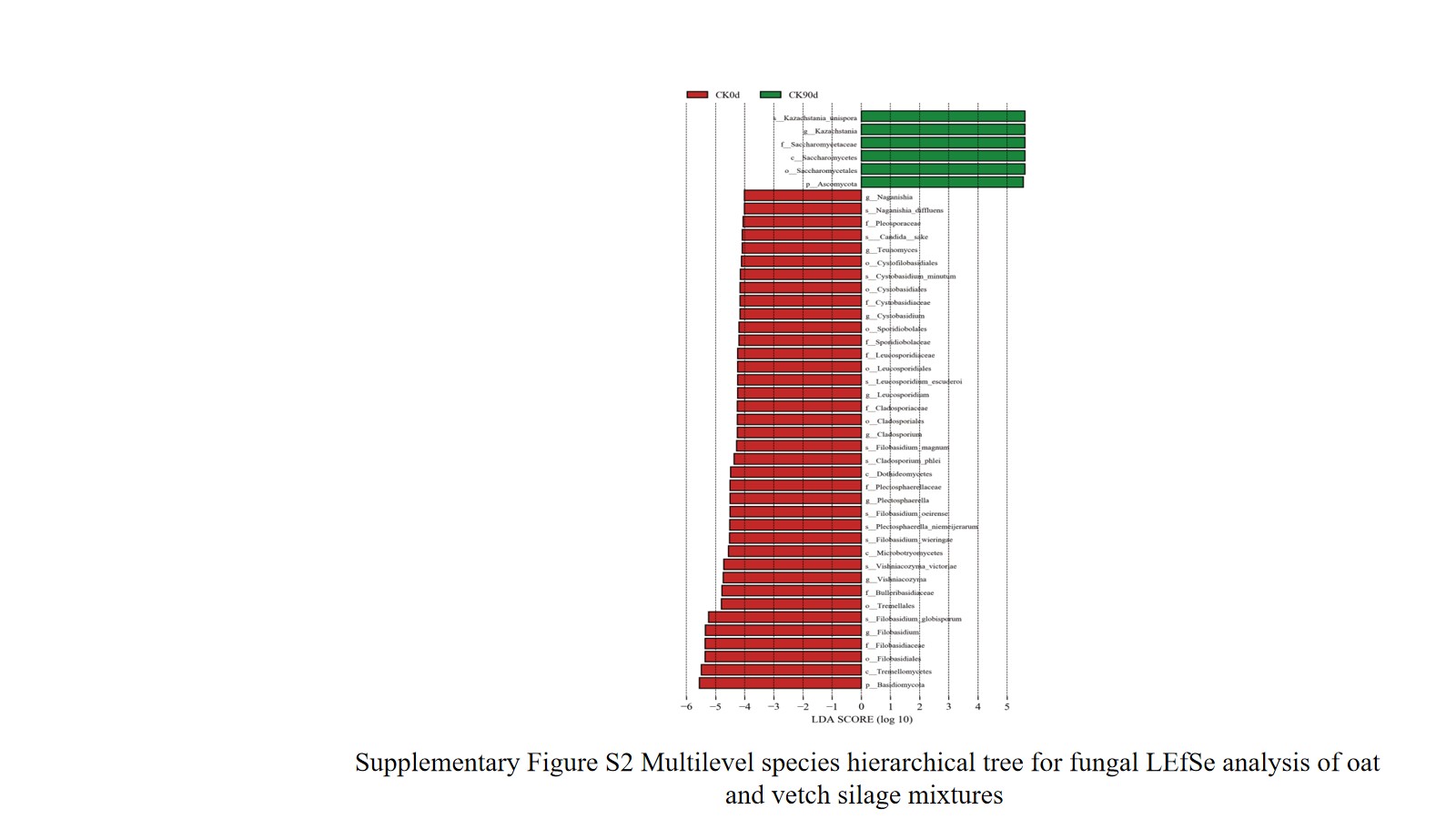

Supplement: Supplementary file 1 [file microorganisms-13-01535-s001.zip › Supplementary Figure S2.jpg]

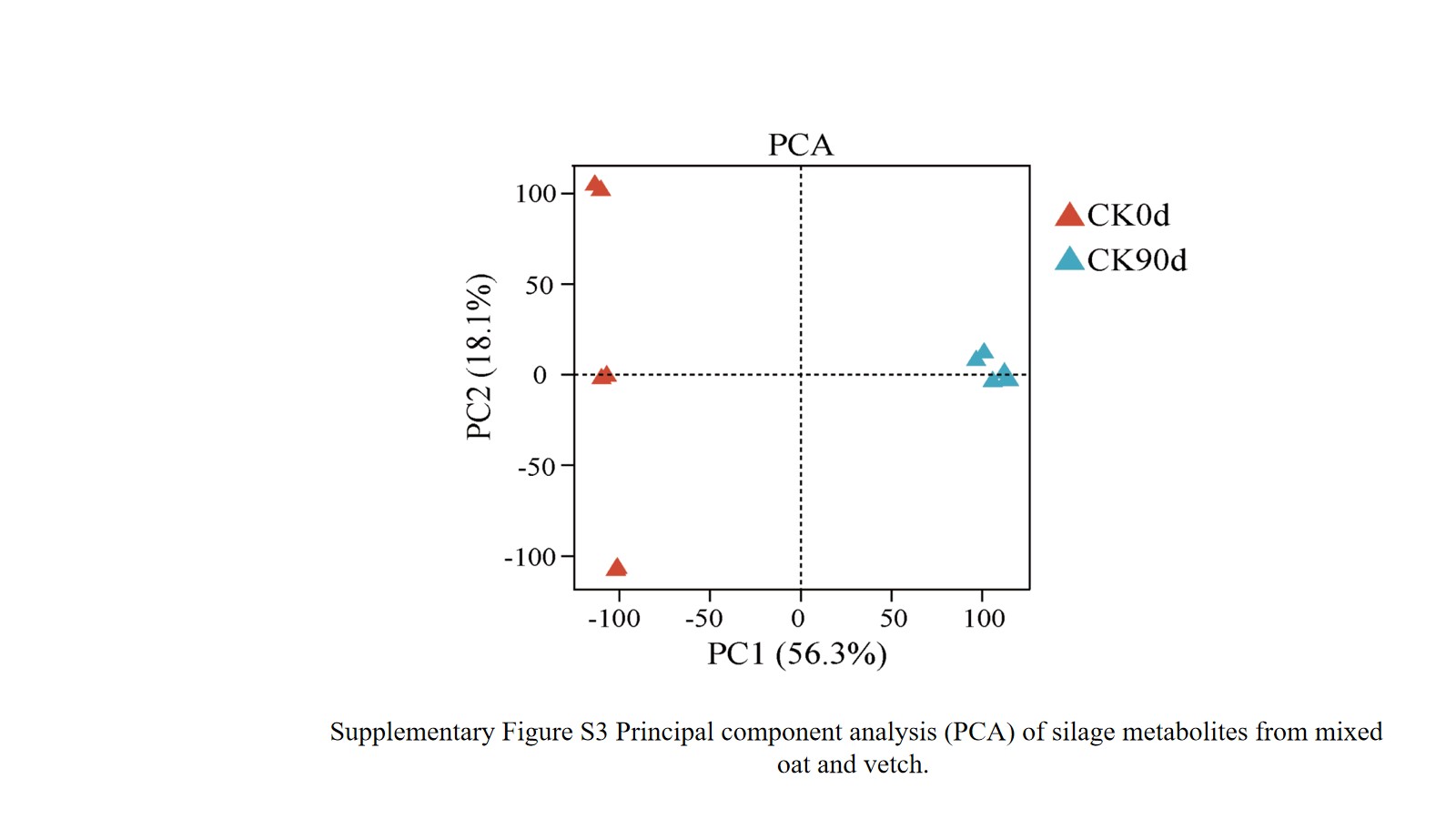

Supplement: Supplementary file 1 [file microorganisms-13-01535-s001.zip › Supplementary Figure S3.jpg]
